# Comparative Evaluation of Post-operative Analgesia Using Visual Analogue Score (VAS) With a Low Dose of Clonidine (0.5 mcg/kg) as an Adjuvant to 0.2% Ropivacaine in Ultrasound-Guided Supraclavicular Block in Upper Limb Surgeries

**DOI:** 10.7759/cureus.104270

**Published:** 2026-02-25

**Authors:** Babita Sheoran, Ramesh Lamba, Kashish Sindhwani, Vishnu Vaish, Monika Sangwan, Mayur Tuteja

**Affiliations:** 1 Pharmacology, Pt. Neki Ram Sharma Government Medical College and Hospital, Bhiwani, IND; 2 General Surgery, Civil Hospital, Bhiwani, IND; 3 Pharmacology, Kalpana Chawla Government Medical College, Karnal, IND; 4 Biochemistry, Shree Guru Gobind Singh Tricentenary (SGT) University, Gurugram, IND; 5 Pathology and Laboratory Medicine, Pt. Neki Ram Sharma Government Medical College and Hospital, Bhiwani, IND

**Keywords:** adjuvant analgesia, clonidine, postoperative analgesia, regional anesthesia, ropivacaine, upper limb surgery, visual analog scale (vas)

## Abstract

Background

Effective pain management during and after surgery is essential for improving patient outcomes. Adding adjuvants like clonidine to local anesthetics enhances block efficacy and improves post-operative analgesia.

Objective

To evaluate the efficacy and safety of adding low-dose clonidine (0.5 mcg/kg) to 0.2% ropivacaine in ultrasound-guided supraclavicular brachial plexus block for upper limb surgeries by assessing the duration of postoperative analgesia, pain intensity, time to first pain complaint, time to requirement of rescue analgesia, and the influence of demographic variables (age and gender) on analgesic efficacy and duration.

Methods

A prospective observational study included 90 patients undergoing upper limb surgery. Group A (n=45) received ropivacaine, while Group B (n=45) received the same with clonidine. Pain was assessed using the Visual Analog Scale (VAS) at 0, 6, 12, and 24-48 hours postoperatively. Primary outcomes were the duration of analgesia, time to the first pain complaint, and VAS scores. Statistical analysis was done using SPSS, version 29 (p<0.05).

Results

Group B showed a significantly longer duration of analgesia (14.33±0.91 hours) compared to Group A (6.96±1.13 hours, p<0.001). VAS scores at 6 and 12 hours were significantly lower in Group B (p<0.001). No significant adverse effects were noted.

Conclusion

Adding clonidine to ropivacaine in supraclavicular brachial plexus blocks significantly prolongs analgesia and reduces pain scores without notable side effects, demonstrating it as an effective strategy for improving patient comfort.

## Introduction

Effective peri-operative pain management is a fundamental goal of modern anaesthetic practice, as inadequately treated pain may lead to increased sympathetic activity, delayed functional recovery, prolonged hospitalisation, and reduced patient satisfaction. Contemporary anaesthesia therefore emphasises techniques that provide optimal analgesia while minimising systemic adverse effects [1]. In this context, regional anaesthesia has gained prominence due to its opioid-sparing benefits, improved postoperative analgesia, and favourable safety profile when compared with general anaesthesia alone.

The supraclavicular brachial plexus block is widely regarded as an effective regional anaesthetic technique for upper limb surgeries, as it provides dense anaesthesia of the upper extremity distal to the shoulder, regarded as the “spinal of the upper limb” [2]. The routine use of ultrasound guidance has significantly improved block success rates and safety by allowing real-time visualisation of neural structures and adjacent vessels, thereby reducing complications such as pneumothorax, intravascular injection, and local anaesthetic systemic toxicity (LAST) [3].

Ropivacaine and patient safety

Ropivacaine, a long-acting amide local anaesthetic, has emerged as a preferred agent for peripheral nerve blocks owing to its reduced cardiotoxicity and neurotoxicity compared to bupivacaine [4,5]. Its lower lipid solubility and stereoselective pharmacology allow for preferential sensory blockade with relatively less motor blockade, facilitating early mobilisation and functional recovery [6]. The use of low-concentration ropivacaine (0.2%) has gained acceptance in regional anaesthesia as it provides adequate analgesia while minimising the risk of LAST, excessive motor block, and delayed rehabilitation, though many fewer studies are available regarding such lower concentrations [7]. However, the duration of postoperative analgesia with low-dose clonidine alone may be limited, prompting the use of adjuvants to enhance block duration without increasing local anaesthetic dose.

Role of clonidine as a low-dose adjuvant

Adjuvants such as opioids, dexamethasone, and alpha-2 adrenergic agonists have been explored to improve the quality and duration of peripheral nerve blocks. Among these, alpha-2 agonists have demonstrated consistent analgesia-prolonging effects. Clonidine, a selective partial alpha-2 adrenergic agonist, exerts its analgesic action through inhibition of norepinephrine release, hyperpolarisation of dorsal horn neurons, attenuation of nociceptive transmission, and local vasoconstriction, which delays systemic absorption of local anaesthetics [8,9].

Several clinical studies have shown that the addition of clonidine to ropivacaine in supraclavicular brachial plexus blocks significantly prolongs the duration of sensory blockade and postoperative analgesia while reducing analgesic requirements [10-12]. Importantly, recent evidence suggests that low-dose clonidine (approximately 0.5 mcg/kg) provides an optimal balance between efficacy and safety. At this dose, clonidine prolongs analgesia without causing clinically significant hypotension, bradycardia, or sedation, which have been associated with higher doses [13,14].

Rationale for low-dose combination

The combination of low-concentration ropivacaine (0.2%) with low-dose clonidine (0.5 mcg/kg) represents a patient-centred, safety-oriented approach to regional anaesthesia. This strategy enhances postoperative analgesia, reduces opioid consumption, maintains haemodynamic stability, and supports early recovery, aligning with enhanced recovery after surgery (ERAS) principles. Limited meta-analyses and comparative studies are available to support the use of clonidine as an effective and safe adjuvant when used in appropriate low doses under ultrasound guidance [15,16].

Aim of the study

The present study was undertaken to evaluate the effect of adding clonidine (0.5 mcg/kg) to 0.2% ropivacaine in ultrasound-guided supraclavicular brachial plexus blocks for upper limb surgeries, with particular emphasis on the duration of postoperative analgesia, pain intensity, and patient safety.

## Materials and methods

This prospective observational study was conducted at Kalpana Chawla Government Medical College and Hospital (KCGMC), Karnal, Haryana, after obtaining approval from the Institutional Ethics Committee. The study adhered to the ethical principles of the Declaration of Helsinki and ICH-Good Clinical Practice guidelines. Written informed consent was obtained from all participants.

The calculated sample size was approximately 85, calculated as:

$N = \frac{Z^{2} \times SD^{2}}{d^{2}}$

where N=number of samples to be collected; Z=standard normal variant (Z=1.96 for 95% confidence interval) SD= standard deviation of the variable (taken from previous study) (47) [11]; d=desired precision =10.

N= $\frac{(1.96)^2 \times (47)^2}{(10)^2}$

N=84.86.

A total of 90 adult patients aged 18-60 years, of either sex, classified as American Society of Anesthesiologists (ASA) physical status I or II, scheduled for elective upper limb surgery under ultrasound-guided supraclavicular brachial plexus block were included in the study to account for possible dropouts. Patients with block failure, known hypersensitivity to study drugs, significant cardiovascular, hepatic, renal, neurological, or psychiatric illness, pregnancy, or chronic analgesic use were excluded.

Group A (45 patients) received 15 ml of ropivacaine (0.2%) with 15 ml of 2% lignocaine+adrenaline, while Group B (45 patients) received the same combination plus 1 ml of 0.5 mcg/kg clonidine.

Participant flow is reported in accordance with the CONSORT (Consolidated Standards of Reporting Trials) Group recommendations. A total of 102 patients were assessed for eligibility during the study period. Twelve patients were excluded: six did not meet the inclusion criteria, three declined participation, and three were excluded due to anticipated block contraindications. There were no intraoperative dropouts, protocol deviations, or losses to follow-up. Data from all 90 participants were complete and analyzed on a per-protocol basis.

Demographic variables, including age, sex, weight, and ASA status, were recorded. Postoperative pain intensity was assessed using the Visual Analog Scale (VAS) [17] at 0, 6, 12, and 24-48 hours. The primary outcome was duration of analgesia, defined as time to first rescue analgesic (VAS>5). Secondary outcomes included time to first pain complaint, incidence of postoperative nausea and vomiting, and adverse drug reactions [18]. Causality assessment of suspected adverse reactions was performed using the World Health Organisation- Uppsala Monitoring Centre (WHO-UMC) scale [19,20].

Statistical analysis was performed using SPSS, version 29 (IBM Corp, Armonk, NY). Continuous variables were assessed for normality using the Shapiro-Wilk test and visual inspection of histograms. Normally distributed data are presented as mean±standard deviation and were compared using the independent Student’s t-test. Repeated VAS pain scores across time intervals were analyzed using repeated measures ANOVA for normally distributed data. Categorical variables, including gender, ASA status, and adverse drug reactions, were analysed using the chi-square test. A two-tailed p-value <0.05 was considered statistically significant.

## Results

Sociodemographic details

Table 1 illustrates that both groups were comparable in terms of age, gender, and weight: The mean age was 37.47±12.79 years in Group A and 37.07±13.10 years in Group B. Group A had 36 men and nine women, while Group B had 35 men and 10 women. The mean weight was 65.93±7.75 kg in Group A and 62.42±5.66 kg in Group B. All participants belonged to ASA physical status I or II, and there was no statistically significant difference between groups in baseline variables.

**Table 1:** Demographic details {1}: Age coding for age group 18-30 years; {2}: age coding for age group 31-50 years; {3}: age coding for age group >50 years.

Variables	Group A		Group B	
Age groups (years)	Frequency (n)	Percentage (%)	Frequency (n)	Percentage (%)
18-30 {1}	16	35.6	17	37.8
31-50 {2}	18	40.0	20	44.4
>50 {3}	11	24.4	8	17.8
Mean age	37.47+12.795		37.07+13.103	
Sex				
Male	36	80	35	77.8
Female	9	20	10	22.2
Total	45	100	45	100
Weight (kg)				
Mean+SD	65.93+7.748		62.42+5.663	

Pain scores across time periods (VAS scores)

**Figure 1:**
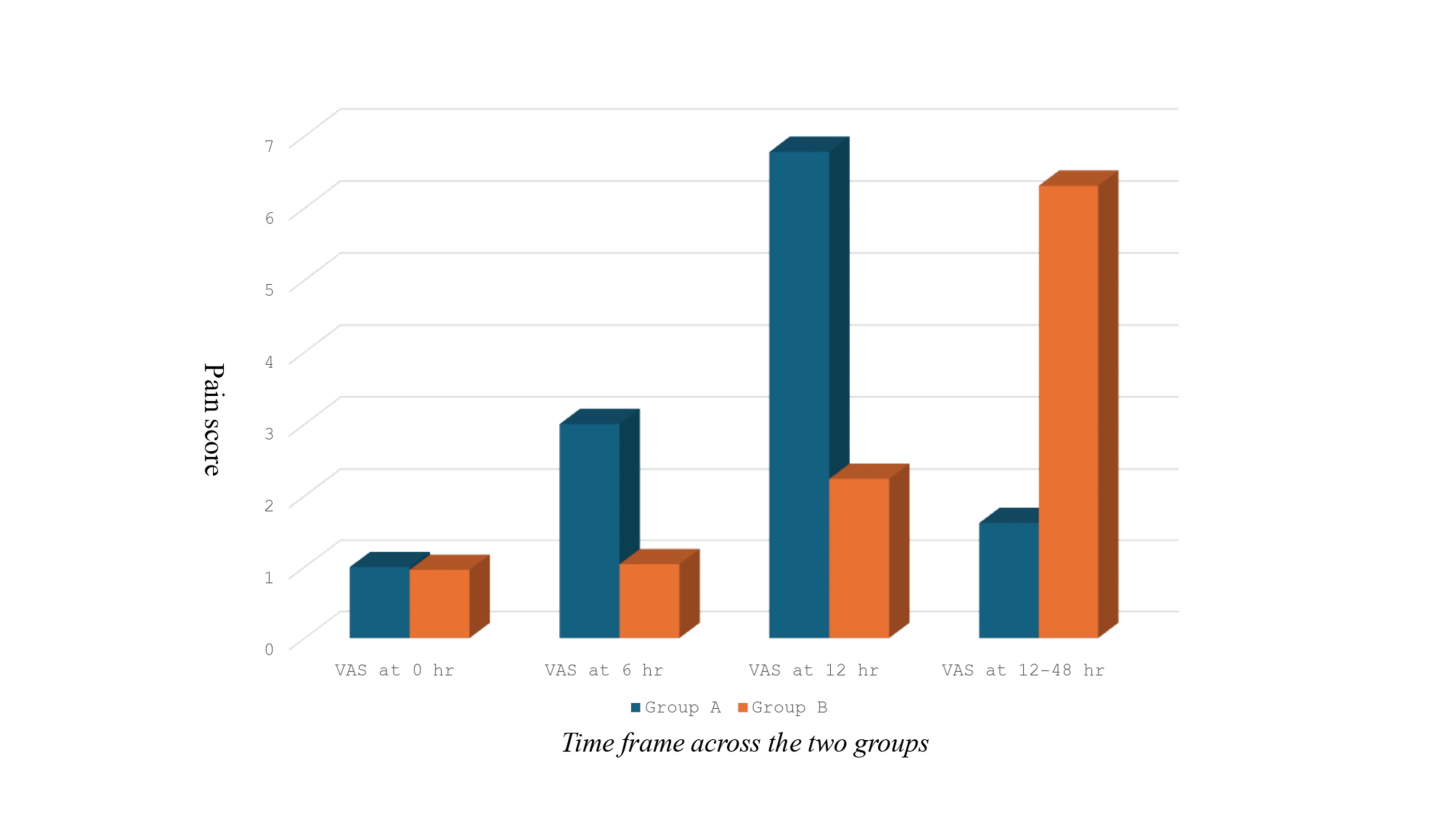
VAS score comparison at different time intervals between two groups VAS: Visual analogue score.

As indicated in Table 2 at 0 hours, pain scores were similar between the groups (Group A: 1.00±0.52 vs. Group B: 0.96±0.48, p=0.352). At six hours, pain scores were significantly higher in Group A (3.00±0.08) compared to Group B (1.04±0.07, p<0.001). At 12 hours, Group A again recorded higher scores (6.78±0.20) versus Group B (2.24±0.15, p<0.001). From 12 to 48 hours, the pain score was higher in Group B (6.31±0.16) compared to Group A (1.62±0.14, p<0.001). Figure 1 compared the VAS scores at different time spans.

**Table 2:** Mean pain scores across different time periods

Pain		Mean	SD	95% confidence		P value
Score (VAS)				interval for mean		
				Lower bound	Upper bound	
0 hour	Group A	1.00	.522	.84	1.16	0.352
	Group B	.96	.475	.81	1.10	
6 hours	Group A	3.00	.078	2.84	3.16	<0.001
	Group B	1.04	.071	.90	1.19	
12 hours	Group A	6.78	.196	6.38	7.17	<0.001
	Group B	2.24	.153	1.94	2.55	
12-48 hours	Group A	1.62	.136	1.35	1.90	<0.001
	Group B	6.31	.155	3.43	4.50	

Post-operative analgesia duration

**Figure 2:**
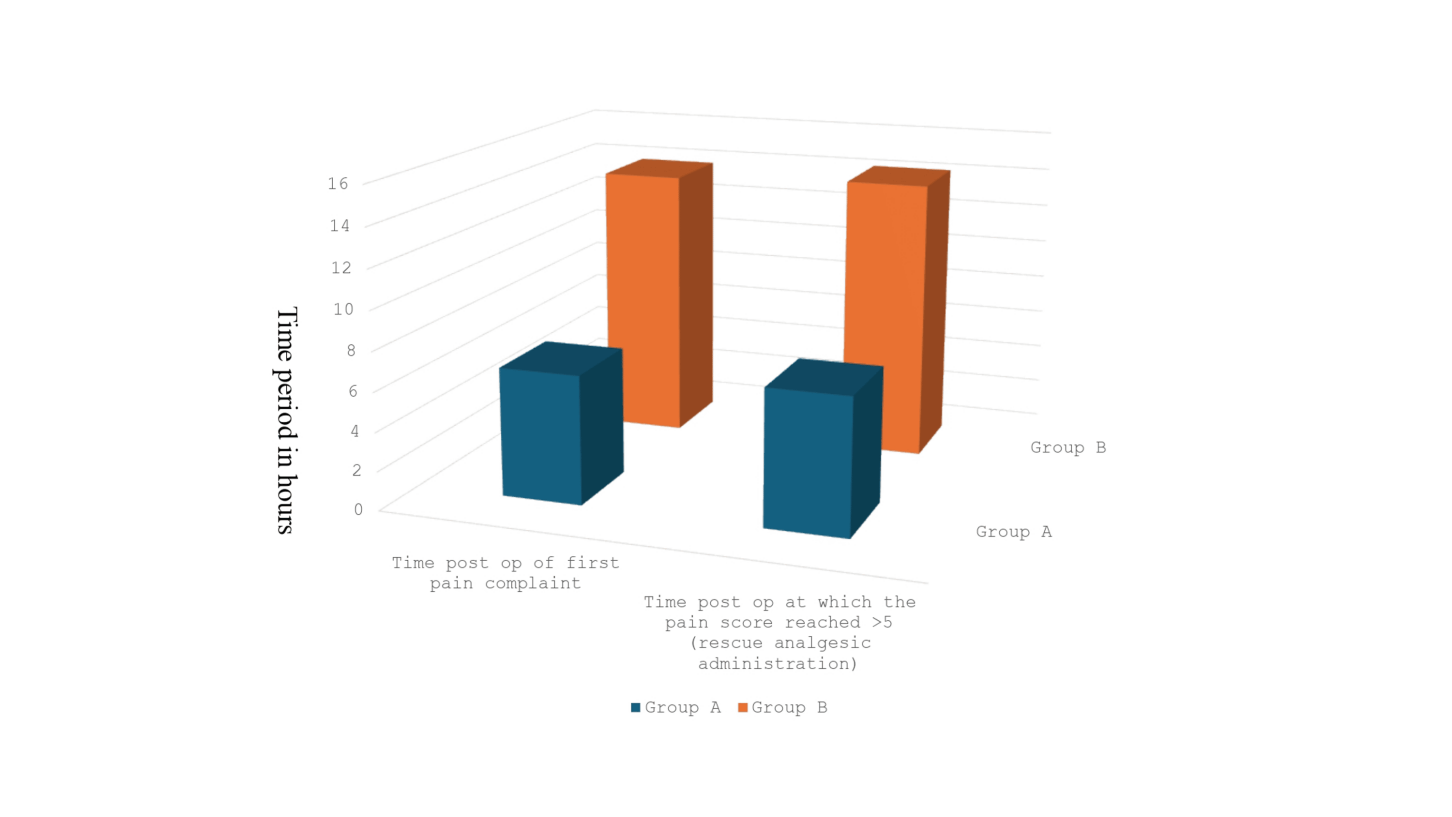
Differences in post-operative analgesia over time

As shown in Figure 2 and Table 3, the mean time for the first pain complaint was 6.60±1.32 hours in Group A and 13.97±0.93 hours in Group B (p<0.001). The time to administer rescue analgesics (VAS>5) was significantly longer in Group B (14.33±0.91 hours) compared to Group A (6.96±1.13 hours, p<0.001).

**Table 3:** Post-operative analgesia time

	Group	Mean	Std. Deviation	Std. Error Mean	P value
Post-op time duration of first pain complain	A	6.600	1.3212	0.1969	<0.001
	B	13.967	0.9256	0.1380	
Post-op time duration when pain score is >5	A	6.9611	1.13247	0.16882	<0.001
	B	14.3267	0.91014	0.13568	

Gender-wise comparison of pain scores

Table 4 demonstrates a statistically significant difference in gender distribution between men and women across both groups. However, comparison of the mean pain scores revealed no statistically significant differences between men and women at the various time intervals assessed.

**Table 4:** Pain score across gender at different time periods

	Sex	N	Mean	Std. deviation	P value
Pain score at 0 hour	F	19	1.11	.315	.043
	M	71	.94	.532	
Pain score at 6 hours	F	19	1.74	.991	0.092
	M	71	2.10	1.123	
Pain score at 12 hours	F	19	4.47	2.389	.468
	M	71	4.52	2.623	
Pain score at 12-48 hours	F	19	4.26	2.353	.281
	M	71	3.89	2.611	
Post-op time duration of first pain complaint	F	19	9.974	4.7389	0.386
	M	71	10.366	3.6430
Post-op time duration when pain score >5	F	19	10.4684	4.67322	0.422
	M	71	10.6908	3.62470	

Statistical significance across variables

Analysis using independent sample tests confirmed a statistically significant difference in post-operative analgesia times and pain scores between the groups, with Group B demonstrating superior analgesia duration and lower pain scores during early postoperative periods (6-12 hours).

Gender-based analysis

**Figure 3:**
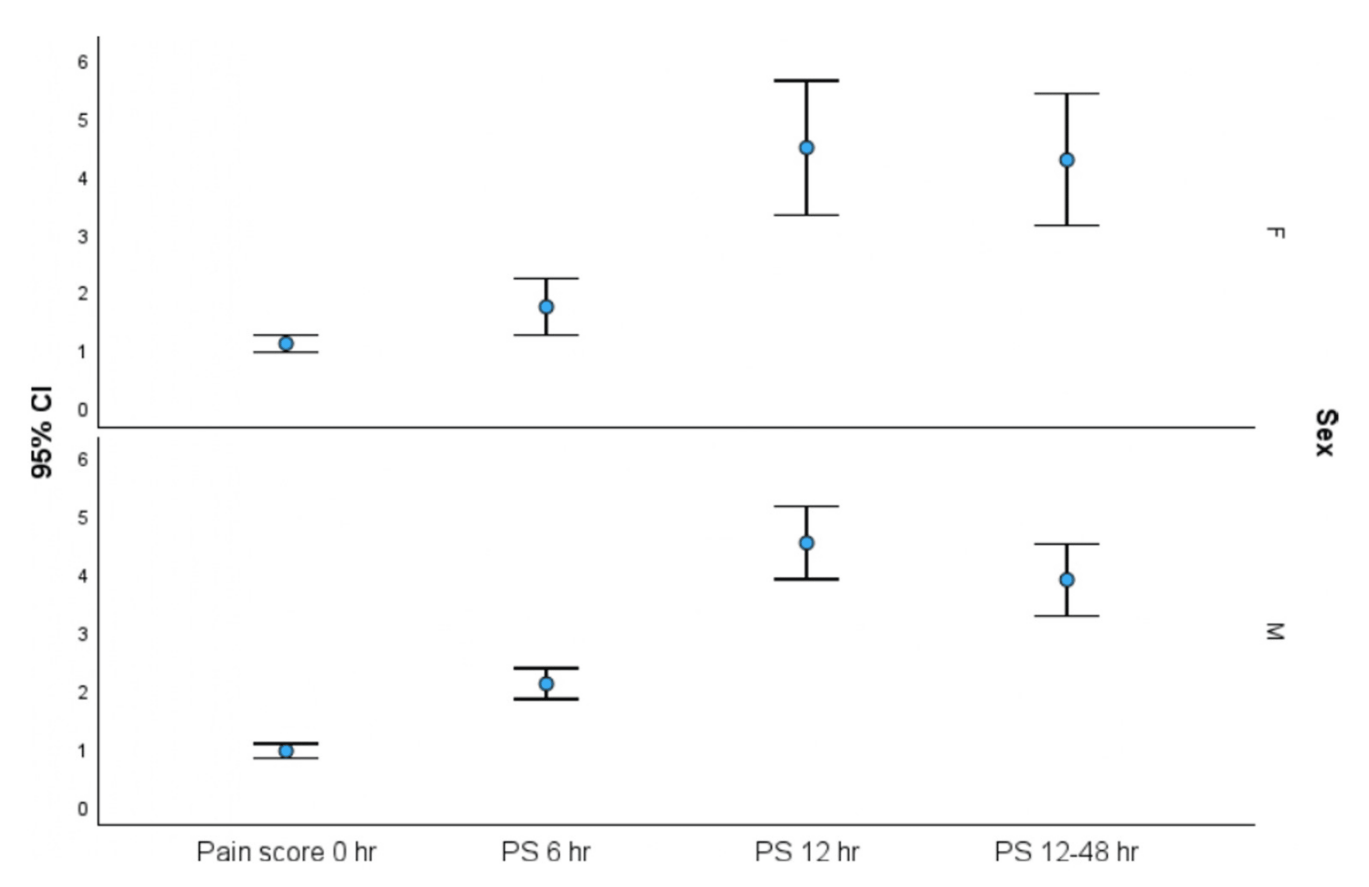
Error plot for pain scores across gender at different time periods.

Figure 3 shows that pain scores and the duration of analgesia did not differ significantly across genders, further validating the uniform efficacy of the drugs across demographic variables.

Secondary outcomes and adverse drug reactions

No significant adverse drug reactions (ADRs) were reported, affirming the safety of clonidine as an adjuvant in these settings. A total of seven adverse drug reactions were observed as shown in Table 5, and four among them were nausea and vomiting and three were hypotension. The WHO-UMC scale for causality assessment was used to monitor and document suspected ADRs, though none were clinically significant. Causality assessment is vital to assess whether the reaction is because of drug alone or other factors are also involved in ADR occurrence, the causality assessment was done using WHO-UMC causality assessment scale and found that the all of ADRs were probable.

**Table 5:** ADR in two groups ADR: Adverse drug reaction.

Parameters	Group A (N=45)		Group B (N=45)	
	No.	%	No.	%
Nausea/vomiting	2	4.4	2	4.4
Hypotension	1	2.2	2	4.4
Sedation	0	0	0	0
Bradycardia/tachycardia	0	0	0	0
Hypertension	0	0	0	0
Pruritus	0	0	0	0
Respiratory depression	0	0	0	0

## Discussion

Regional anaesthesia has emerged as a preferred option for patients undergoing surgeries, particularly those with multiple comorbidities. This is due to its ability to maintain patient consciousness while minimising the need for airway management and ventilation, which reduces the risk of side effects and interference with vital centres. Over the past decade, ultrasound-guided peripheral nerve blocks have become the standard of care among anaesthesiologists, allowing for more accurate and effective anaesthesia. To improve the efficacy of these blocks, various adjuvants have been introduced, among which clonidine, a selective alpha-2 adrenergic agonist, has gained considerable attention for its ability to prolong analgesia [8,9].

In our study, we assessed the effectiveness of ropivacaine at lowest concentration possible combined with clonidine at a comparative lower dose in supraclavicular brachial plexus blocks under ultrasound guidance. Supraclavicular blocks are particularly effective for upper extremity surgeries, as they target the nerve trunks at a level where nearly all the sensory and motor innervation of the upper limb converges, allowing for rapid onset and reliable anaesthesia. The addition of clonidine to ropivacaine has been shown in various studies to prolong the duration of post-operative analgesia, with significant adverse effects. Our study aimed to focus on comparative evaluation of post operative analgesia at much lower doses keeping an eye on patient safety.

Patient characteristics

In terms of patient demographics, our study did not find any significant differences between the two groups in terms of age, sex, and weight, which is consistent with previous studies. The mean age in both groups was approximately 37 years, and the male-to-female ratio was also similar between the groups, although there were slightly more men in both groups. These baseline parameters were in line with studies by Gupta et al. [20], Patil et al. [11] and Rohan et al. [21], which reported similar findings. This ensures that the results observed in study can be generalised to a broader patient population.

Drug dosage and composition

Our study utilised ropivacaine (0.2%) designated as group A. Group B received an additional adjuvant, clonidine (0.5 mcg/kg), which was well within the safe dosage range. The studies by Chakraborty et al. [22] and Sirohiya et al. [23] also demonstrated that small doses of clonidine (30 mcg) significantly prolonged the duration of analgesia without causing major side effects, which is consistent with our findings though the use of bupivacaine instead of ropivacaine in the above-mentioned studies does not correlate the findings much. The addition of clonidine was aimed at enhancing the duration of post-operative analgesia without compromising safety.

Duration of analgesia

The key finding of our study was the significant difference in the duration of post-operative analgesia between the two groups. The mean duration of analgesia in Group A (ropivacaine alone) was 6.96 hours, whereas in Group B (ropivacaine+clonidine), it was 14.33 hours, a difference of approximately 7.37 hours. This extended duration of analgesia in Group B is in agreement with previous studies, including those by Gupta et al. [20] and Chakraborty et al. [22] who also found that the addition of clonidine to local anaesthetics significantly prolonged the analgesic effects but at much higher concentration of ropivacaine (0.75%) [24], though the lower dose of clonidine in this study is another feather to the cap. The prolonged analgesia is likely due to clonidine's action on the alpha-2 adrenergic receptors, which inhibit the release of norepinephrine and reduce the excitability of nociceptive neurons. This leads to a more sustained analgesic effect.

VAS scores and analgesic requirements

Our study also compared the visual analogue scale (VAS) pain scores across different time intervals, and we observed a significant reduction in pain scores in Group B compared to Group A, particularly at 12 hours post-surgery and beyond. Group A required rescue analgesia at 12 hours, whereas Group B maintained significantly lower pain scores throughout the observation period. These findings are in agreement with previous studies by El Saied et al. [10] and Ali et al. [12], which demonstrated that the addition of clonidine significantly reduced VAS scores and decreased the requirement for rescue analgesics. However, unlike the present study, those studies employed higher concentrations of ropivacaine (0.75% and 0.5%, respectively) and did not observe a statistically significant difference in certain outcome parameters, highlighting the advantage of achieving effective analgesia even with lower concentrations of ropivacaine when combined with clonidine.

Side effects

Regarding side effects, our study found that both groups experienced minor adverse reactions, including nausea, vomiting, and hypotension, although the incidence was not statistically significant. These side effects were more frequently observed in Group B, but the low dose of clonidine used in our study (0.5 mcg/kg) likely minimised the risk of severe side effects. Studies such as those by Bernard and Macaire [13] and Büttner et al. [14] reported higher incidences of hypotension and bradycardia with higher doses of clonidine, but such reactions were not observed in our cohort. This highlights the safety of the lower dose of clonidine in prolonging analgesia without causing significant hemodynamic instability.

The strength of our study lies in its robust sample size of 90 patients, which is larger than many similar studies, thereby providing more reliable and generalisable results. Additionally, our study was the first of its kind in the northern region of India, providing valuable data for evaluating the impact of clonidine on post-operative analgesia duration at such a low dose. Furthermore, the inclusion of VAS pain scores at multiple time points allowed for a comprehensive analysis of the analgesic effects and the duration of pain relief.

The limitation of our study was the lack of stratification of the female population across the groups, which could influence the results. Gender differences in pain perception and analgesic requirements have been well documented, and future studies should ensure an equal distribution of male and female participants to better assess the influence of gender on analgesic outcomes.

The absence of randomisation limits causal inference, and the observed associations should therefore be interpreted cautiously. Although efforts were made to standardise technique and postoperative assessment, unmeasured perioperative variables such as individual pain perception, psychological factors, and surgical variability may have influenced outcomes. Additionally, the study was conducted at a single centre, which may limit external generalisability to other institutions with different patient populations, anaesthetic practices, or perioperative protocols.

Follow-up was limited to the early postoperative period; therefore, long-term analgesic outcomes and delayed adverse effects could not be evaluated. Finally, although ASA I-II patients were intentionally selected to ensure safety and homogeneity, this restricts applicability of findings to higher-risk populations.

Future randomised controlled trials with multicentre participation, blinding, and extended follow-up are required to confirm these findings and strengthen causal interpretation.

## Conclusions

This study provides strong evidence for the efficacy of adding clonidine to ropivacaine in prolonging the duration of analgesia in supraclavicular brachial plexus blocks, setting the standardisation of the dose of clonidine along with ropivacaine for future use. The addition of clonidine resulted in a significantly longer period of post-operative pain relief, with minimal side effects observed. These findings are consistent with other studies and support the use of clonidine as a safe and effective adjuvant in peripheral nerve blocks. Future studies with larger, gender-stratified populations and long-term follow-up are needed to further validate these results and explore the potential of this combination for broader clinical use.
